# Evidence of novel fine-scale structural variation at autism spectrum disorder candidate loci

**DOI:** 10.1186/2040-2392-3-2

**Published:** 2012-04-02

**Authors:** Dale J Hedges, Kara L Hamilton-Nelson, Stephanie J Sacharow, Laura Nations, Gary W Beecham, Zhanna M Kozhekbaeva, Brittany L Butler, Holly N Cukier, Patrice L Whitehead, Deqiong Ma, James M Jaworski, Lubov Nathanson, Joycelyn M Lee, Stephen L Hauser, Jorge R Oksenberg, Michael L Cuccaro, Jonathan L Haines, John R Gilbert, Margaret A Pericak-Vance

**Affiliations:** 1 Hussman Institute for Human Genomics, University of Miami Miller School of Medicine, 1501 NW 10 Ave, M-860, Miami, FL 33136, USA; 2 Department of Neurology, School of Medicine, University of California, 220 Montgomery St., 5th Fl., San Francisco, CA 94143-0248, USA; 3 Center for Human Genetics Research, Vanderbilt University, 519 Light Hall, 2215 Garland Ave. South, Nashville, TN 37232-0700, USA

**Keywords:** AUTISM, CGH, CNV, GABA, NRXN1

## Abstract

**Background:**

Autism spectrum disorders (ASD) represent a group of neurodevelopmental disorders characterized by a core set of social-communicative and behavioral impairments. Gamma-aminobutyric acid (GABA) is the major inhibitory neurotransmitter in the brain, acting primarily via the GABA receptors (GABR). Multiple lines of evidence, including altered GABA and GABA receptor expression in autistic patients, indicate that the GABAergic system may be involved in the etiology of autism.

**Methods:**

As copy number variations (CNVs), particularly rare and *de novo *CNVs, have now been implicated in ASD risk, we examined the GABA receptors and genes in related pathways for structural variation that may be associated with autism. We further extended our candidate gene set to include 19 genes and regions that had either been directly implicated in the autism literature or were directly related (via function or ancestry) to these primary candidates. For the high resolution CNV screen we employed custom-designed 244 k comparative genomic hybridization (CGH) arrays. Collectively, our probes spanned a total of 11 Mb of GABA-related and additional candidate regions with a density of approximately one probe every 200 nucleotides, allowing a theoretical resolution for detection of CNVs of approximately 1 kb or greater on average. One hundred and sixty-eight autism cases and 149 control individuals were screened for structural variants. Prioritized CNV events were confirmed using quantitative PCR, and confirmed loci were evaluated on an additional set of 170 cases and 170 control individuals that were not included in the original discovery set. Loci that remained interesting were subsequently screened via quantitative PCR on an additional set of 755 cases and 1,809 unaffected family members.

**Results:**

Results include rare deletions in autistic individuals at JAKMIP1, NRXN1, Neuroligin4Y, OXTR, and ABAT. Common insertion/deletion polymorphisms were detected at several loci, including GABBR2 and NRXN3. Overall, statistically significant enrichment in affected *vs*. unaffected individuals was observed for NRXN1 deletions.

**Conclusions:**

These results provide additional support for the role of rare structural variation in ASD.

## Background

Autism spectrum disorders (ASD) represent a group of neurodevelopmental disorders characterized by a core set of social-communicative and behavioral impairments. There is abundant evidence for a strong genetic contribution to ASD, with concordance rates among MZ twins ranging from 60% to 90% [1]. It is now evident that the underlying genetic architecture of autism is highly complex, with numerous genomic loci contributing to overall risk. Efforts to identify specific risk loci have met with some success, and variants in several genes and genomic regions having been implicated to date. These loci include *SHANK3*, *CNTNAP2*, *NLGN4X*, *pCDH10*, *16p11.2*, *NRXNa1*, *SYNGAP*, *SEMA5A*, and *AVPR1A*, among others [2-6]. Evidence for the role of common variation in ASD risk was reported within the CDH9/CDH10 region of chr5 on risk [7]. Despite these important advances, phenotypic and genetic heterogeneity have made it challenging to unravel the underlying causes of ASD, and a substantial component of this disorder's genetic etiology remains to be discovered.

### Copy number variation and ASD

A number of studies have implicated copy number variations (CNVs) in ASD [8-15]. In addition to specific structural mutations, global enrichment in rare and/or *de novo *CNVs has been reported within affected *vs*. unaffected individuals (for example [5,7-15]). To date, studies examining copy number variation in ASD have relied heavily on array technologies that were originally designed for the purpose of conducting SNP-based genome-wide association studies (GWAS). This is primarily due to the fact these data already exist (that is they were obtained for genotyping purposes). These GWAS arrays typically place a limit on the resolution at which CNVs can be reliably detected at 10 to 50 kb or larger (contingent upon the array and analysis parameters), although smaller events can be detected in certain circumstances. The use of custom designed comparative genomic hybridization (CGH) arrays, combined with a targeted, candidate-gene oriented strategy, can extend the ability to reliably detect variation below 10 kb. As it is evident that structural DNA variation with potential relevance to pathological phenotypes can and does exist at sub-10 kb scales (for example [16]), we sought to examine a subset of autism candidate regions at high resolution. The GABA receptor family of genes and genes within biological pathways associated with GABA are attractive candidates for autism and ASD. GABA is the primary inhibitory neurotransmitter in the human brain, and it is known to assume both excitatory and inhibitory roles during early development stages [17,18]. Previously our group has reported evidence of both linkage and association with ASD at the GABA receptor genes in the 15q11-13 region [19,20]. In addition, differential expression of GABA receptors has been observed in ASD cases [21-23], and rare coding variants in GABRB3 have been found that segregate with ASD [24]. In light of the above evidence, we chose to examine 24 GABA receptors and 19 additional GABA-related genes for evidence of sub-5 kb structural variation that might contribute to the autism/ASD risk. We further extended our candidate gene set to include an additional 19 genes and regions that had been previously implicated in autism (Table 1). These loci were either recognized ASD candidates or directly related to ASD candidates through biological function or sequence homology. Targeted genes include members of the Neurexin and Neuroligin gene families, the CDH9/CHD10 region on chromosome 5p, the 16p11.2 regions, CNTNAP2, OXT, OXTR, MECP2, and SHANK3. While most of these candidate genes and regions have been previously examined for structural variation in the context of GWAS array analysis, these loci have not been systematically targeted with custom designed high resolution aCGH in a population of ASD and control individuals to survey fine-scale structural variation.

**Table 1:** Targeted genes and regions (hg18 coordinates)

Gene/locus	Chr	Start	Stop
GABRD	chr1	1940703	1952050
ALDH9A1	chr1	163898073	163934524
GAD2	chr10	26545600	26633493
GABARAPL1	chr12	10256756	10266991
GABR CLUSTER→GABR-A/B/G	chr15	24339787	25451729
ABAT	chr16	8675928	8785933
GABARAPL2	chr16	74157750	74169280
ZDHHC7	chr16	83565573	83602642
GABARAP	chr17	7084462	7086477
LOC649186	chr19	61520464	61521066
DBI	chr2	119840974	119846592
GAD1	chr2	171381446	171425905
SLC32A1	chr20	36786519	36791429
GABRR3	chr3	99188217	99236521
ZDHHC3	chr3	44941665	44992618
JAKMIP1	chr4	6106385	6253183
GABRG1	chr4	45732544	45820839
GABRA2	chr4	45946463	46087178
GABRA4	chr4	46615674	46690337
GABRB1	chr4	46728336	47123202
SLC6A7	chr5	149549713	149570828
GABRB2	chr5	160648014	160907708
GABRA6	chr5	161045548	161061690
GABRA1	chr5	161206983	161258992
GABRG2	chr5	161427295	161515106
GABRP	chr5	170143343	170173628
ALDH5A1	chr6	24603176	24645414
GABBR1	chr6	29631387	29708839
GABRR1/GABRR2	chr6	89944691	90081673
GABBR2	chr9	100090187	100511300
GABRE	chrX	150872252	150893807
GABRA3	chrX	151087186	151370486
GABRQ	chrX	151557293	151572481
SV2A	chr1	148140546	148159156
SLC12A5	chr20	44091245	44122196
SLC38A3	chr3	50217709	50233408
SLC6A1	chr3	11009456	11055935
SLC6A11	chr3	10832917	10955146
SLC6A12 & SLC6A13	chr12	167240	245958
CHR5 Peak Association Region	chr5	24400928	27150205
CD38	chr4	15389029	15459804
OXT	chr20	3000266	3001162
OXTR	chr3	8767095	8786300
BDNF	chr11	27631325	27707500
EN2	chr7	154943585	154950287
MECP2	chrX	152940458	153016323
SHANK3	chr22	49459936	49518504
MBD1	chr18	46045698	46064286
MBD2	chr18	49916985	50016488
MBD3	chr19	1527678	1543652
MBD4	chr3	130632483	130641542
NRXN1	chr2	49993980	51150855
NRXN2	chr11	64130222	64247236
NRXN3	chr14	77528262	79502905
FOXP2	chr7	113404235	114320494
CNTNAP2	chr7	145444386	147749019
chr16p11.2	chr16	29478715	30278715
NLGN1	chr3	174785171	175483810
NLGN2	chr17	7252226	7263903
NLGN3	chrX	70278940	70308868
NLGN4-Y	chrY	15144026	15464921
NLGN4-X	chrX	5818083	6156706
**Positive and negative controls (common polymorphisms)**			
Deletion/duplication	chr1	109988334	109993086
Deletion/duplication	chr3	99893514	99897268
Deletion	chr3	194260213	194265258
Deletion/duplication	chr10	132799241	132802636
Duplication	chr16	4710525	4715494

## Methods

### Ethical approval

This research was carried out in accordance with the World Medical Association's Declaration of Helsinki. Participants in the study were ascertained under applicable IRB protocols of the John P. Hussman Institute for Human Genetics (HIHG), University of Miami, Miller School of Medicine, Vanderbilt University Medical Center, and University of California, San Francisco (UCSF) Medical School. Following a description of the study, informed consent was obtained for each individual or, where appropriate, their guardian.

### Ascertainment and DNA extraction

Individuals were recruited as part of our ongoing family-based recruitment via support groups, advertisements, and clinical and educational settings. Written informed consent was obtained from parents for all minor children and those who were unable to give consent. In addition, we obtained assent from all participants of the appropriate developmental and chronological age. Core inclusion criteria were as follows: (1) between 3 and 21 years of age, (2) a clinical diagnosis of ASD, (3) an expert clinical determination of ASD diagnosis using DSM-IV criteria supported by the Autism Diagnostic Interview (ADI-R) [25,26], and (4) an IQ equivalent > 35 or developmental level >18 months as determined by the Vineland Adaptive Behavior Scale (VABS) [27]. A best estimate diagnostic determination was based on review by a three-person panel, including experienced clinical psychologists and a pediatric medical geneticist. This determination included ADI-R results. In those instances where an ADI-R was not available (*n *= 86; six were parents, 67 were lost to follow-up, refused to complete the interview, or withdrew, and 13 are in progress), a best-estimate diagnosis was assigned by this panel using all available clinical information including clinician summaries, caregiver report, and medical records. Following review of case materials and discussion, panel members reached a consensus. We excluded participants with severe sensory problems (for example visual impairment or hearing loss), significant motor impairments (for example failure to sit by 12 months or walk by 24 months), or identified metabolic, genetic, or progressive neurological disorders. Family history and pedigree information was collected in a standard semi-structured interview from a knowledgeable informant, frequently the mother. Additional clinical data were also collected by reviewing available medical and psychiatric records of affected individuals.

The majority of control participants were recruited by the HIHG at the University of Miami and included 565 children between the ages of 3 and 21 years. Participants with developmental, behavioral, or neurological conditions were excluded, as well as those with first-degree relatives with such disorders. We obtained consent from all participants or, in the case of minors, their parents. Participants meeting these criteria provided a saliva sample. A knowledgeable informant, usually the mother, completed the Social Communication Questionnaire to screen for potential ASDs [28]. The above controls were supplemented with additional set of 65 individuals obtained from the UCSF School of Medicine. Blood samples were obtained from self-reported Non-white Hispanics, ranging from 15 to 37 years of age and reporting no history of chronic disease.

For the CNV discovery stage of the project, DNA from whole blood and/or saliva (Oragene kit) was collected from 173 Caucasian non-Hispanic and Caucasian Hispanic autism cases and 185 racially matched control individuals (≤37 years of age) with no known signs of ASD (matched backgrounds were confirmed with Eigenstrat clustering [29]). As described below, data from some samples were excluded from further analysis based on laboratory QC metrics, resulting in a final sample set of 168 cases and 149 controls. CNV loci that were molecularly confirmed and trended towards significance were subsequently tested within a larger sample set of 2,564 individuals, consisting of 755 affected (728 Caucasian/non-Hispanic, 17 Asian, 7 Hispanics, 3 African-Americans) and 1,809 unaffected individuals who were family members of the 755 cases.

DNA from both blood and saliva sources was extracted using the Autopure (Gentra) system according to the manufacturer's standard protocols. For a subset of six control individuals for which DNA extracted from both blood and saliva sources was available, we ran replicate CGH arrays on both blood and saliva DNA in order to look for any systematic differences evident between the two sources. Although batch effects attributable to numerous experimental factors have been previously noted for CNV studies, quality metrics and total CNV events called indicated no significant DNA-source effects within this CGH-based study. Specifically, Nexus 4.1 (Biodiscovery) noise metrics and CNV copy call numbers showed no systematic differences between blood and saliva groups (Additional file 1: Table S1).

### Target gene selection

In addition to the set of GABA-related gene targets forming the primary focus of this study, a set of 19 additional ASD candidates were selected based on several criteria, including a recently completed GWAS [7]. The list included both a set of genes previously implicated in the ASD literature, as well as genes known to interact biologically and/or exhibit significant homology to those genes. As not all putative and/or established ASD loci could be accommodated on the array, the final prioritized list was arrived at by consensus among study collaborators.

### CGH array design

We designed a custom 244 k Agilent comparative genomic hybridization (CGH) array targeting our regions of interest. Array hybridization probes were chosen using Agilent earray software https://earray.chem.agilent.com/earray/, with a preference for catalogue probes, where available. Probe design was based on coordinates from the hg18 build of the human reference sequence. Collectively, our probes spanned a total of approximately 11 Mb with an average probe density of one unique probe every 200 nucleotides. Given that most standard CNV calling approaches require four to five consecutive significant probes to identify an aberrant region, our coverage theoretically allows for the detection (on average) of CNVs 800 bp or larger. The GABA family of receptors poses particular challenges for hybridization-based copy number assays. Due to the level of sequence homology resulting from the ancestral relationships among GABA genes, increased background probe noise within conserved regions was anticipated. In an effort to mitigate the influence of assay noise on the CNV calling algorithm, we employed triplication of all internal experimental probes as well as running technical array replicates of a subset of 40 individuals to aid in identifying and excluding problematic regions.

### Array processing

Genomic DNA was fragmented, enzymatically labeled, hybridized to arrays, and washed according to the manufacturer's (Agilent) standard protocols. Briefly, 0.75 μg of extracted DNA from a single hybridization reference individual (sample NA10851, available from the US National Institute of General Medical Sciences (NIGMS) Human Genetic Cell Repository) was labeled with Cy3 dye (following recommendation of [30]). An equal amount of extracted DNA from experimental samples was enzymatically labeled with Cy5 dye. Labeled samples for each experimental individual was combined with labeled reference DNA, hybridized to the CGH array for 40 h at 65°C, and washed to remove non-hybridized fragments prior to imaging.

### Imaging and analysis

All arrays from this study were imaged using a single Molecular Devices GenePix 4000B microarray scanner, processed with Agilent Feature Extraction 9.5 and analyzed with Nexus (Biosdiscovery) software version 4.1. Preliminary analysis was conducted using Rank Segmentation (an extension of the circular binary segmentation algorithm [31] as implemented in Nexus 4.1. Final results presented in this manuscript are based on the Nexus Rank segmentation algorithm with the gain/loss LogR threshold set to 0.25, maximum inter-probe distance at 25 kb, and the (default) minimum of five probes required per called CNV event.

### Quality control

As indicated above, each probe was replicated three times (each at a randomly distributed location) on the CGH array and fluorescent signal intensities were averaged prior to subsequent analysis. For detection of common sample handling errors, markers on the X chromosome were examined to ensure observed sample sex was consistent with the original sample manifest. In addition, sex chromosome markers, as a proxy for true-positive controls, a set of five highly polymorphic CNV locations reported in the Database of Genomic Variation http://projects.tcag.ca/variation/ were used to evaluate the ability of our array design (and associated processing technique) to detect structural DNA variation at scales of 5 kb and below (Table 1 bottom). Because the selected loci harbored reported minor allele frequencies of 15% and above, each of the minor structural alleles was expected to be observed, with high probability, one or more times within our sample of 317 individuals. All five of the targeted CNVs were readily detected within our sample population. Although it was observed as variant in our sample set, one of the five targets chosen from the database, hg18 locus *chr16:4710525-4715494*, was found upon further examination to harbor extreme high repetitive content making the reliability of hybridization results questionable. We therefore do not recommend its use as a positive control locus in future experiments.

To identify potential outlier samples (with respect to sample quality) total CNV calls per individual were rank sorted. A clear demarcation point was observed at approximately 60 total CNV calls per individual, with a relatively small number of samples exhibiting significantly higher numbers of CNV calls. We suspected that the excess number of calls associated with these arrays was attributable to poor sample quality or processing issues; as evidence of this, the Nexus 4.1 quality metric (a measure of probe-to-probe variation across contiguous probes) among these individuals was elevated compared to that of the non-excluded set. Collectively, our quality criteria resulted in a subset of 41 samples being excluded from all subsequent analysis.

### Statistical analysis of CNV calls

Initial CNV calls output from Nexus 4.1 rank segmentation algorithm were converted to compatible format for loading into PLINK [32]. For the purpose of analysis, PLINK provides the option of handling heterogeneous CNV calls at the same genomic locus by treating each unique CNV start and stop coordinate as separate markers. Any copy number gain (or loss) that overlaps the position of a marker assumes a value (0, 1, 2, 3, or 4-plus copies) for the marker. After initial processing in PLINK to produce a .ped file with CNV markers, logistical regression analysis was conducted via R scripting. Results of logistic regression analysis are provided in Additional file 2: Table S2. Regression results corresponding to -LOG10 (*P *value) ≥ 3 were evaluated as candidates for additional analysis. For analysis of the larger family-based dataset, the GENMOD procedure (SAS vers8.1) was run on the dataset and a generalized estimating equation (GEE) was employed to account for the relationships between the parents and siblings (unaffected) and cases. GEE allows for specification of a correlation matrix of the between-subject relatedness of participants belonging to the same pedigree [33] and is commonly used in the context of genetic epidemiology studies to produce robust variance estimates that otherwise would likely be too small/conservative in the presence of relatedness between observations [34]. For each of the candidate genes evaluated by GEE at the validation stage of the study, Bonferroni correction was used to correct for the number CNV positions examined within the candidate. Global CNV burden was assessed using the mperm option in PLINK to perform a global permutation test (10,000 permutations) on segment number, total kilobase difference, and average kilobase difference.

### Molecular confirmation of CNV calls

Prioritized loci were validated by real-time quantitative PCR using TaqMan chemistry (a full list of target locations are provided in Additional file 3: Table S3). Assays were performed in quadruplicate, as recommended by the manufacturer (Applied Biosystems **{Life Technologies, Carlsbad, CA}**), reactions were run on the ABI-7900HT real-time PCR system and analyzed using CopyCaller software v.1.0 (Life Technologies, Carlsbad, CA). Regions were chosen for validation based on several criteria, including their potential for biological relevance and logistic regression scores (Additional file 2: Table S2). CNVs spanning known exons were given the highest priority, followed by those including regions of evolutionary conservation, as evidenced by interspecies sequence homology (per UCSC Genome Browser, build 36.1, March 2006, conservation tracks). Some predicted copy number variable regions that exhibited statistically significant scores were nevertheless excluded from further analysis because data from technical replicates indicated poor replicability for calls at these regions. Examination of these loci indicated that they tended to exhibit extreme GC content, repetitive element content, or extensive homology to other areas of the genome likely due to common ancestry and subsequent evolutionary conservation (data not shown). In addition to the above criteria, CNV regions were excluded from follow-up if they exibited 75% or greater overlap with known copy number variable regions reported in DGV http://projects.tcag.ca/variation/; however, exceptions were made in cases where the variants encompassed exons within candidate genes and instances where gained or loss alleles were discordant in case/controls. All loci meeting these criteria subjected to TaqMan CNV assays (Applied Biosystems) for further interrogation in additional cases and controls.

## Results and discussion

A total of 24 GABA receptors, 19 GABA-associated coding genes, and 19 additional autism candidates were targeted for aCGH assessment (Table 1). After exclusion of outlier samples, a total of 168 case and 149 control individuals were examined by CGH arrays in the initial discovery set. In all, 6,143 CNV events calls were identified across our tested population, representing 1,223 potentially unique variations. The overall number of CNV events did not exhibit significant differences between ASD affected and unaffected individuals, although both the average and total kilobases impacted did show statistical significance (perm *P *< 0.001; 10,000 permutations; one-sided), consistent with previous reports indicating pathogenic CNVs tend to be larger in size and over-represented in affected individuals (for example [35]). The full list of CNV calls from the Nexus Rank Segmentation algorithm is provided in Additional file 4: Table S4. The logistic regression analysis of case and control allele frequencies were used, in conjunction with additional biological annotation criteria, to prioritize a subset of regions for confirmation via a second molecular platform, TaqMan copy number quantitative PCR assays (Applied Biosystems). In addition to the ASD candidate loci prioritized for follow-up, five control genomic regions, representing known polymorphic locations with minor allele frequencies >10%, were also targeted with TaqMan assays to test the sensitivity of our laboratory technique. The 29 targeted regions and five control loci targeted for follow-up are detailed in Additional file 3: Table S3, along with confirmation results. A diagram of the work flow for discovery and validation is provided in Additional file 5: Figure S1. The sample set used for molecular confirmation and additional population screening was comprised of the individuals in which the CNVs were initially observed (for confirmation), along with an additional 170 Caucasian cases and 170 unaffected ethnically-matched controls that were not present in the original discovery set (Table 2). A subset of independently confirmed loci that remained interesting was run on a larger set of 755 case and 1,809 unaffected family members. GEE analysis results for these data, accounting for relationships between individuals, are provided in Table 3.

**Table 2:** Taqman case/control confirmation set data

Indel region (hg18 coordinates)	LOCUS	Estimated size (bp)	Case (gain)	Control (gain)	Case (loss)	Control(loss)
Previously described rare events						
chr3:8,749,197-8,795,876	OXTR^a^	46679	1	0	1	1
Novel rare events						
chr16:8,666,148-8,704,012	ABAT	37864	0	0	1	0
chr2:50,948,094-51,013,968	NRXN1-probeA	65874	0	0	1	1
	NRXN1-probeB		0	0	1	0
chr3:50,207,690-50,243,268	SLC38A3	35578	0	0	5	8
chr4:6,158,503-6,162,618	JAKMIP1	4115	0	0	1	1
chrY:15,135,574-15,474,858	NLGN4Y	339284	0	0	1	0

^a^OXTR loci was previously reported in [33] but was examined here within a novel group of individuals.

**Table 3:** GEE results from family-based validation dataset (*n *= 2,564, 755 affected and 1,809 unaffected individuals)

	Parameter	Z PR	*P *value	OR	Lower limit	Upper limit
GABBR2_1^a^						
	Deletion	0.3100	0.7582	1.14	0.500	2.587
	Duplication	-2.1800	0.0292	0.72	0.542	0.968
	Event	-1.9800	0.0476	0.76	0.579	0.997
NRXN1_1^a^						
	Deletion	2.3000	0.0215	2.75	1.161	6.508
	Duplication	1.1500	0.2515	3.24	0.435	24.085
	Event	2.5300	0.0114	2.81	1.262	6.258
NRXN1_2^a^						
	Deletion	2.1400	0.0320	2.33	1.075	5.059
	Duplication	-0.9700	0.3309	0.34	0.040	2.963
	Event	0.8500	0.3959	1.40	0.644	3.040

^a^Contains deletion or duplication significant at *P *< 0.05. Only loci demonstrating statistical significance are shown here.

Targeted CNV call events were confirmed with quantitative PCR at 13 of 29 loci, 45%. This suggests a minimum false-positive rate (at the locus level) of 55% (addressed below).

### Phenotypic evaluation

Medical records were examined for a subset of individuals harboring rare CNV events at loci of interest (ABAT, NRXN1, JAKMIP1, GABBR2, NLGN2, OXTR). We examined salient phenotypic and medical history characteristics for these individuals to look for commonalities among carriers of CNVs at particular loci. Characteristics examined included developmental abnormalities in speech and motor development, learning disabilities, and evidence of dysmorphologies. Additional co-morbidities (for example irritable bowel syndrome, asthma) and neuropsychiatric features were also noted. No obvious correspondence between aberrant CNV state and specific phenotypic traits was observed. A summary of available phenotypic and medical characteristics for the individuals examined is provided in Additional file 6: Table S5.

In addition to these rare variants, common structural polymorphisms were observed and validated at multiple loci, including NRXN3, CNTNAP2, GABBR2, SLC38A3, and NLGN2. Of these more frequent events, only the GABBR2 locus approached nominal significance (GEE; *P *= 0.029; OR = 1.16; 95% CI: 0.7-1.93) in our larger validation set, but the result was not significant at α = 0.025 after correction for multiple testing (Table 3).

The role of structural variation, particularly rare and *de novo *CNVs, in autism has been established in a number of previous studies. Here we examined a set of autism candidate loci using high-resolution aCGH analysis. Our principal objective was to detect structural variants at these loci that may have been potentially missed by other screening platforms, such as SNP-based arrays. Among the limitations impacting our study was the presence of a high false-positive rate (in excess of 50%) in our initial aCGH discovery set. The most likely explanation for this elevated false-positive rate is that the majority of genes in our study, including both GABA and non-GABA-related targets (for example MBD1-MBD4), belong to families of homologous genes which can lead to aberrant probe hybridization. As the majority of loci in our study belonged to homology groups, we could not formally test for this effect. In addition, the modest overall sample numbers used in this study limited our ability to accurately estimate population allele frequencies for rarer events. Even with these complicating factors, several promising events were detected and independently confirmed by quantitative PCR. Rare structural variations were detected at ABAT, NLGN4-Y, NRXN1, and JAKMIP1 (Table 2). Most notable among these results, statistically significant enrichment of deletions (GEE; *P *= 0.0215; OR = 2.75; 95% CI: 1.161-6.5; α = 0.025) in affected *vs*. unaffected individuals was observed at NRXN1 within our larger family-based validation set with after correction for multiple testing (Table 3).

We observed and confirmed an approximate 4 kb deletion in a female autistic indvidual within the JAKMIP1 gene that removes two exons. JAKMIP1, a member of a family of microtubule interacting proteins, is known to interact with GABBR1 protein [36] and knockdown of its expression via siRNA results in increased GABBR2 expression, suggesting a possible role for JAKMIP1 in GABBR2 regulation. Misregulation of JAKMIP1 has also been observed in lymphoblastoid cell lines from autistic individuals [37]. We note, however, that no significant enrichment for the deletion was observed in our larger validation set. More detailed characterization of these deletions in the affected and unaffected individual is warranted before further inference can be made, as the precise boundaries of the deletion events may be different in case and control individuals.

**Figure 1:**
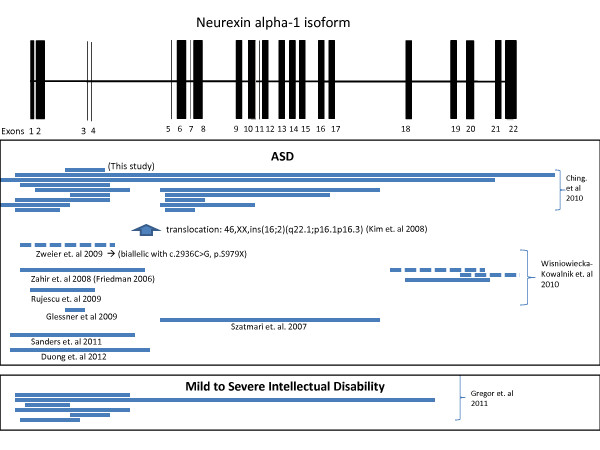
**A deletion of approximately 70 kb encompassing two exons (3,4) from the NRXN1 gene (alpha-1-isoform)**. Previously reported NRXN1 CNV events in ASD cases are shown mapped below [38-40,42-49]. A subset of NRXN1 structural variants reported in individuals with mild to severe intellectual disability is also depicted [49]. Solid lines indicate deletions (majority) and dashed lines indicate duplication events.

A large (approximately 70 kb) deletion encompassing two exons of the NRXN1 genes was detected in an autistic male (Figure 1). NRXN1 is a neuronal adhesion molecule that has been implicated in a number of neurological disorders, including autism (for example [38-41]. Suggestive SNP association results at the NRXN1 locus were reported in a large collaborative study, but genome-wide significance was not reached [7]. Significant enrichment was previously reported for rare structural events [42]. The removal of this exonic region within an autistic male in our dataset, along with the detection of significant enrichment of NRXN1 deletions in cases *vs*. unaffected individuals in this study, provides further evidence for NRXN1 as an ASD risk locus. Furthermore, the location of our CNV, along with the placement of previously reported NRXN1 events (Figure 1), suggest that disruptions of the alpha form of NRXN1. The beta form of NRXN1, which is transcribed from a secondary downstream promoter, is less frequently observed to be disrupted by CNV in ASD cases. Combined with the aforementioned genetic association results, our data suggests that both rare and common variants may be contributing to ASD risk at this genomic location.

A deletion of the entire Neuroligin 4Y gene within an autistic male was also observed and confirmed. In the larger dataset, the deletion was observed in the original individual's unaffected father as well as within an unaffected male sibling. Two additional unaffected carriers (unrelated) were also observed in the larger dataset. Interestingly, however, we observed two duplication events within the larger validation set, both of which were present in unrelated ASD individuals. Neuroligin 4Y, which resides on the non-recombining portion of the Y chromosome in modern humans was, in the evolutionary past, allelic with Neuroligin 4X (*NLGN4X*) gene. Several studies have previously implicated NLGN4X in autism (for example [50-52]). Nucleotide mutations in NLGN4Y have been reported in an autistic male and his father, who possessed learning disabilities [53]. While there is currently no data regarding the functional status and possible role of NLGN4Y, EST evidence from the NCBI database suggests that NLGN4Y retains a molecularly active promoter and is expressed in the brain. A recent report demonstrated a potential role for some pseudogenes in regulating the expression of their parent or source genes by providing decoy targets for miRNAs [54]. It is possible that, through this or some other mechanism, NLGN4Y is involved in regulating the expression of the NLGN4X locus in males. Our finding of copy number aberrations of this gene in autistic individuals suggests that the locus warrants further investigation.

We observed a deletion at the ABAT gene locus within multiple ASD individuals, one in our discovery dataset and a second case ASD case in the validation set. Further examination of the locus within the larger, family based set revealed 7 of 755 affected individuals carrying the deletion and five (of 1,809) unaffected individuals (unrelated to the seven affected individuals). Although we observed a trend of enrichment in affected vs. unaffected individuals in our dataset, statistical significance was not met at α = 0.05. The observed trend, however, suggests that ABAT locus, and the GABA system as a whole, warrant further scrutiny for their potential role in ASD risk. ABAT is involved in the catabolism of GABA, has previously been implicated in autism by genetic association, although replication of the association was not observed in the small validation dataset (*n *= 91 autism trios) [55]. In the one deleted individual from our dataset for which CGH array data was available at the locus, the ABAT deletion removes exon 1 of the gene (Additional file 7: Figure S2). We note that the nearby A2BP1 and GRIN2A loci, which have also been implicated in autism [55], were not included in our deleted region (Additional file 8: Figure S3).

We also observed a previously reported deletion in the OXTR receptor in a single male ASD individual [56]. A smaller duplication region in OXTR was also detected in a separate ASD case.

In addition to the rare copy number events described above, we also observed and confirmed common copy number polymorphisms at SLC38A3, GABBR2, NRXN3, SHANK3, and NLGN2 (Table 2). Among these, only duplication events in GABBR2 achieved nominal statistical significance at α = 0.05, but this result failed to hold up after multiple correction for multiple testing. Furthermore, the relatively high level of background noise of assays targeting this locus makes us approach the GABBR2 finding with caution. Additional examination of the locus through alternative platforms (for example long-read high throughput sequence technologies) will be necessary before further conclusions can be drawn.

## Conclusions

These data further indicate the importance of structural variation in ASD risk and provide additional evidence that rare variants at multiple genomic loci are potentially contributing to this common neurodevelopmental disorder. In particular, we report statistically significant enrichment of rare exonic deletions in NRXN1 in autistic *vs*. non-autistic individuals. The detection and confirmation of structural variants below the 10-50 kb typically afforded by SNP-based GWAS arrays indicate that aCGH remains an important complementary method for CNV detection.

## Availability of supporting data

The dataset(s) supporting the results of this article are included within additional supplemental files.

## Abbreviations

aCGH: Array-based Comparative Genomic Hybridization; ADI-R: Autism Diagnostic Interview; ASD: Autism Spectrum Disorders; BP: Base Pair(s); CGH: Comparative Genomic Hybridization; CNV: Copy Number Variation; DNA: Deoxyribonucleic Acid; DSM-IV: Diagnostic and Statistical Manual of Mental Disorders IV; GABA: Gamma-aminobutyric acid; GABR: Gamma-aminobutyric acid receptors; GEE: Generalized Estimating Equation; GWAS: Genome Wide Association Study; HIHG: Hussman Institute of Human Genomics; IQ: Intelligence Quotient; MZ: Monozygotic; NIGMS: National Institute of General Medical Sciences; PCR: Polymerase Chain Reaction; UCSF: University of California, San Francisco; VABS: Vineland Adaptive Behavior Scale.

## Competing interests

The authors declare that they have no competing interests.

## Authors' contributions

DJH, MPV, JLH, and JRG conceived study. ZMK, BLB, HNC, PLW, and LN performed experiments. DJH, KLH, GWB, DM, and JMJ analyzed experimental data. SJS, LN, JML, and MLC evaluated clinical data. SLH, JRO, and MPV contributed samples and reviewed manuscript. DJH wrote the manuscript. All authors read and approved the final manuscript.

## Supplementary Material

Additional file 1 **Table S1**. Examination of Noise metric and call rates of blood and spit derived DNA extractions from same individuals. Click here for file

Additional file 2 **Table S2**. Click here for file

Additional file 3 **Table S3**. Validation Targets. Click here for file

Additional file 4 **Table S4**. Click here for file

Additional file 5 **Figure S1**. Outline of experimental work flow. Click here for file

Additional file 6 **Table S5**. Click here for file

Additional file 7 **Figure S2**. A 40 kb deletion removing the first exon of the ABAT gene. Click here for file

Additional file 8 **Figure S3**. Larger genomic region of ABAT exonic deletion. Although the start 5' flank point of the deletion has not been localized, intervening probes between ABAT and A2BP1 genes indicates that the deletion terminates a significant distance away from either A2BP1 or GRIN2A, both which have been implicated in autism. Click here for file
