# Supplementary material for: Evidence of novel fine-scale structural variation at autism spectrum disorder candidate loci
Source: Mol Autism. 2012 Apr 2;3:2. doi: 10.1186/2040-2392-3-2 (PMC3352055; doi:10.1186/2040-2392-3-2)

Experimental Workflow

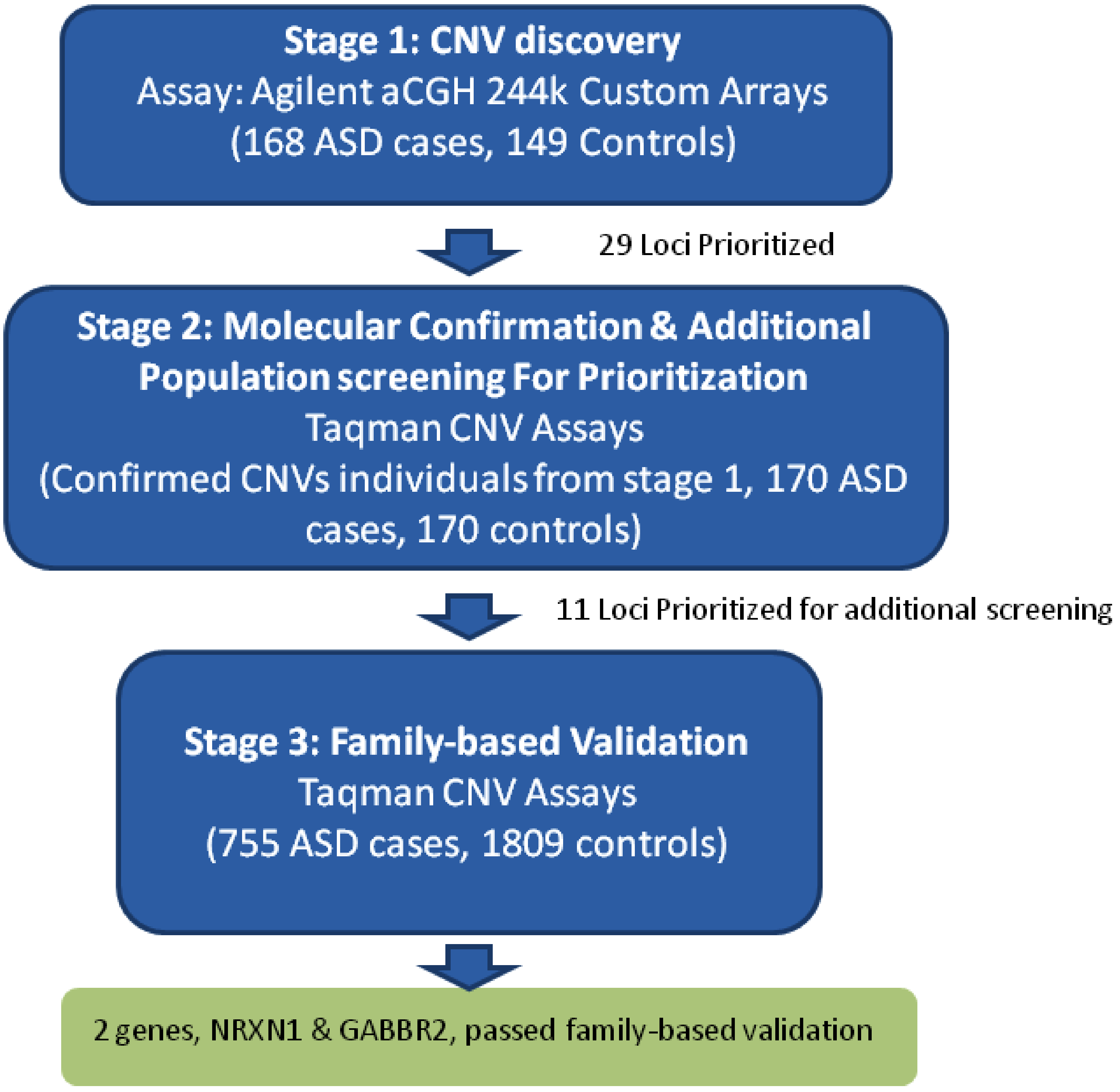

Supplement: Additional file 5 — Figure S1. Outline of experimental work flow. [file 2040-2392-3-2-S5.PDF]
