# Supplementary figures and images for: Evidence of novel fine-scale structural variation at autism spectrum disorder candidate loci
Source: Mol Autism. 2012 Apr 2;3:2. doi: 10.1186/2040-2392-3-2 (PMC3352055; doi:10.1186/2040-2392-3-2)

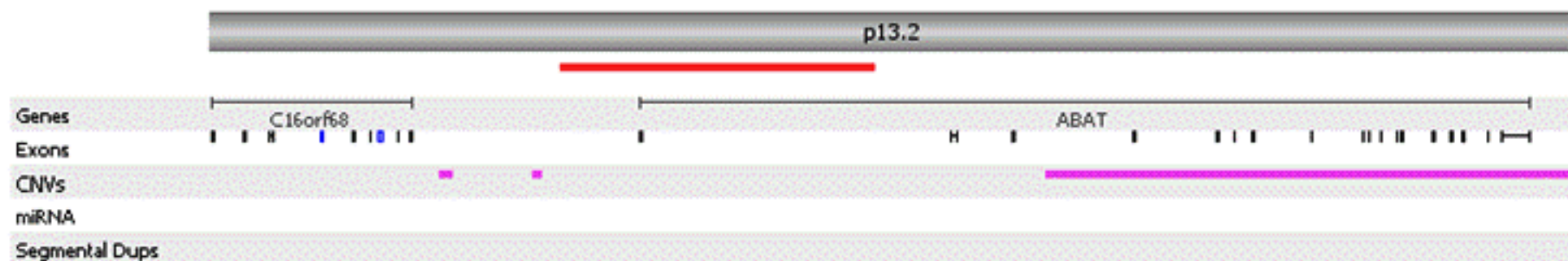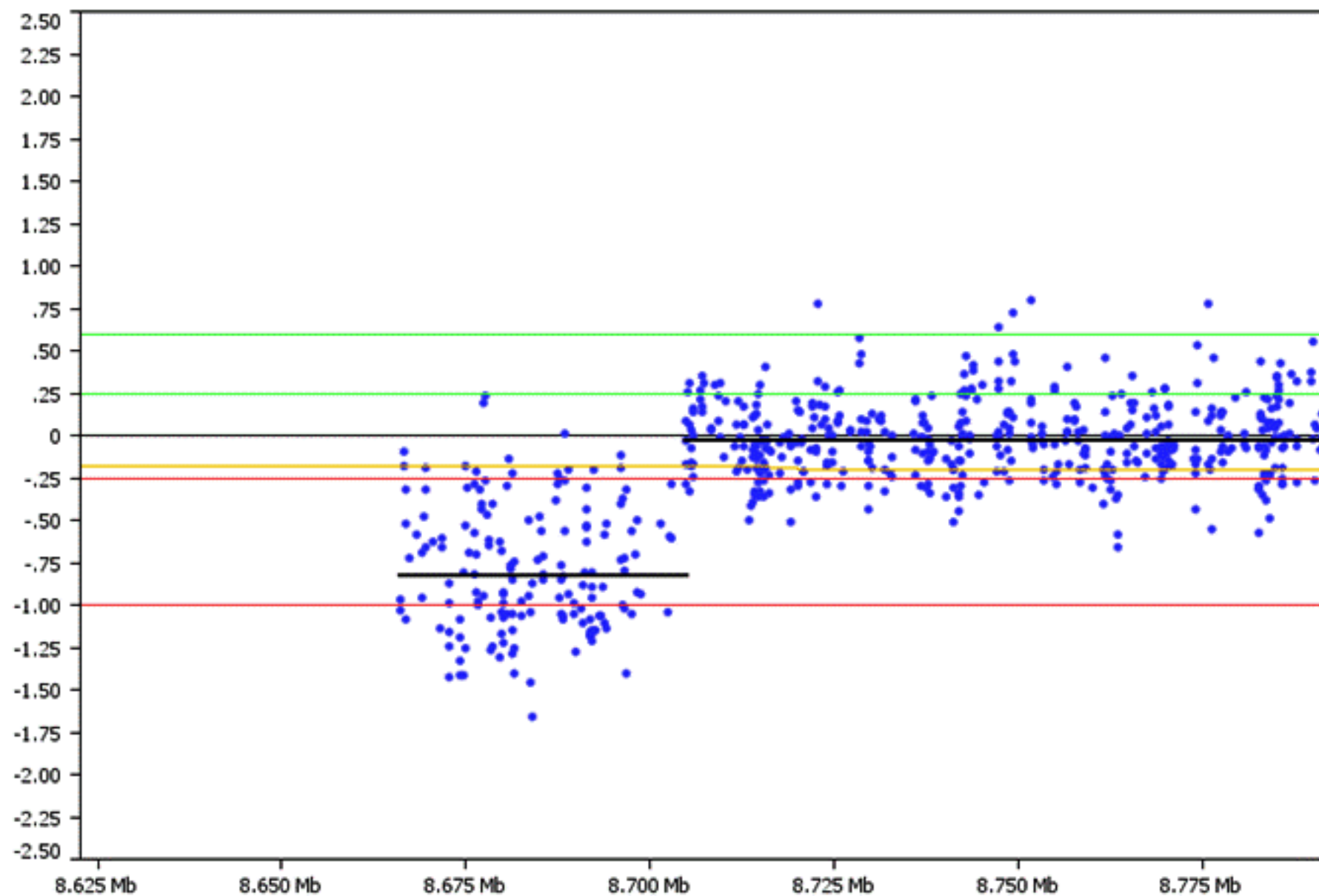

Supplement: Additional file 7 — Figure S2. A 40 kb deletion removing the first exon of the ABAT gene. [file 2040-2392-3-2-S7.PDF]

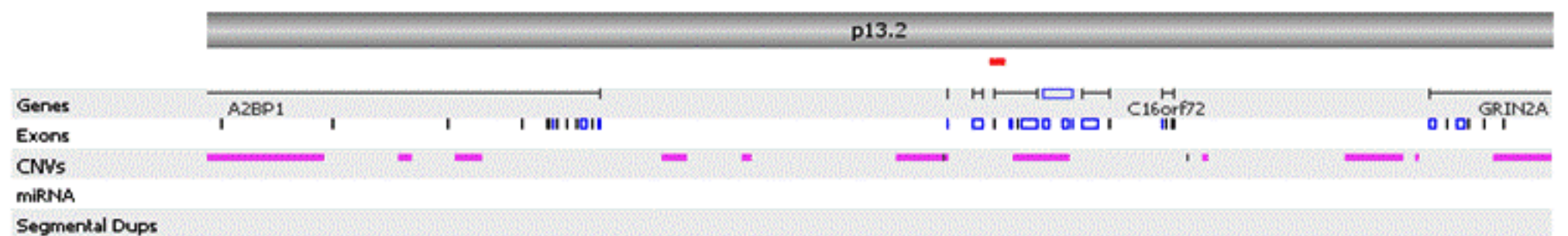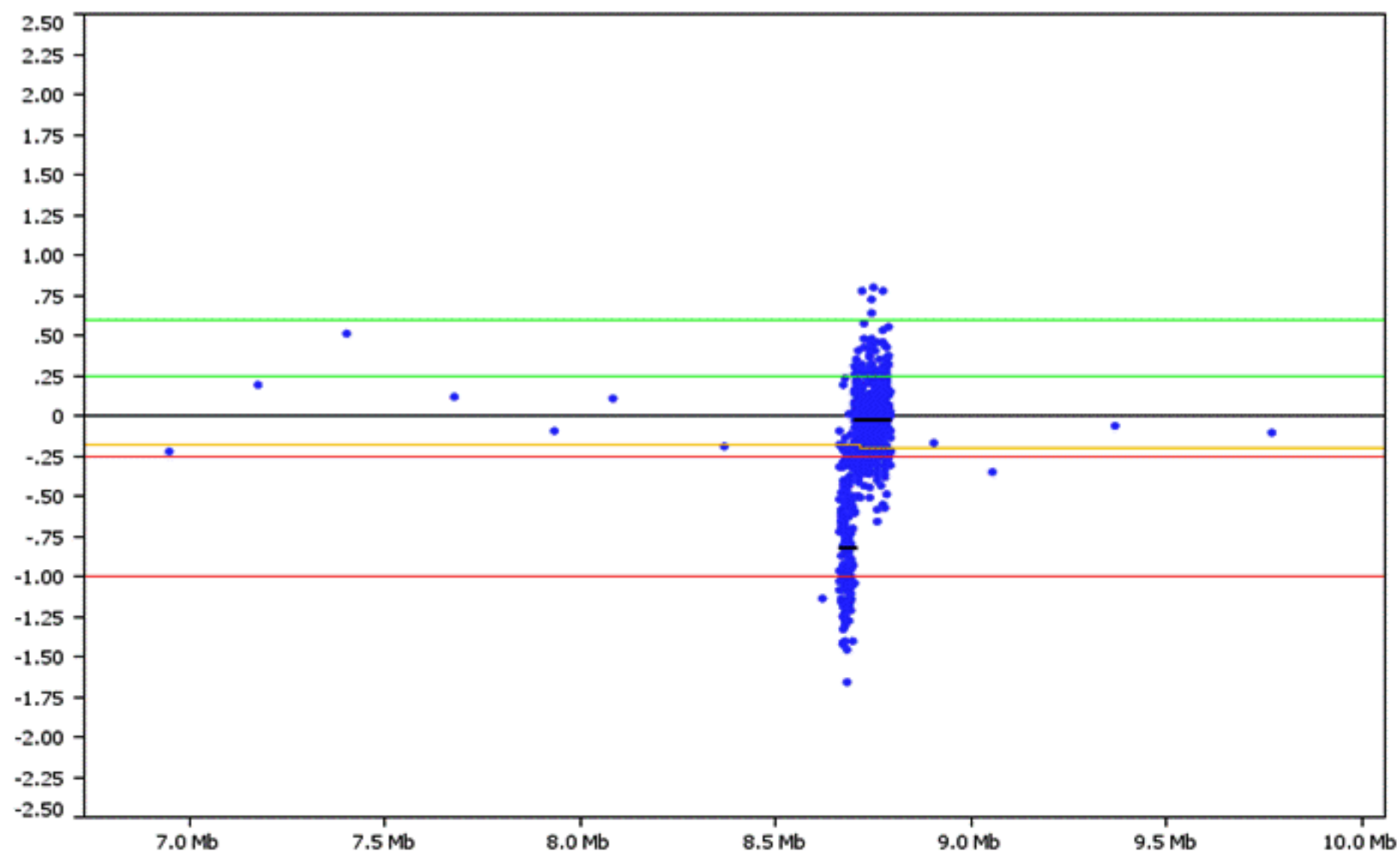

Supplement: Additional file 8 — Figure S3. Larger genomic region of ABAT exonic deletion. Although the start 5' flank point of the deletion has not been localized, intervening probes between ABAT and A2BP1 genes indicates that the deletion terminates a significant distance away from either A2BP1 or GRIN2A, both which have been implicated in autism. [file 2040-2392-3-2-S8.PDF]
